# The Effect of a Low-Energy and Low-Glycemic Diet on Adipose Tissue Metabolism and Energy Expenditure in Women with Excess Body Weight

**DOI:** 10.3390/nu17233789

**Published:** 2025-12-03

**Authors:** Ewa Lange, Ewelina Pałkowska-Goździk

**Affiliations:** Department of Dietetics, Institute of Human Nutrition Sciences, Warsaw University of Life Sciences (SGGW-WULS), 159 C Nowoursynowska Street, 02-776 Warsaw, Poland

**Keywords:** low-glycemic index diet, low-energy diet, obesity

## Abstract

**Background/Objectives**: Data indicate that a low-glycemic index diet may be an effective nutritional approach to improve cardiometabolic parameters and support weight loss in obese individuals. The purpose of the study was to evaluate and compare the effects of a free-choice low-GI diet and a low-energy diet in women with excessive body weight on the value of anthropometric parameters, selected indices of lipid, carbohydrate, and fat tissue metabolism, and alterations in resting metabolic rate (RMR). **Methods**: Forty-six women were randomly assigned to either a low-GI diet (LGI) or a low-energy diet (LED) for 12 weeks. Dietary habits and anthropometric parameters (waist circumference, body weight, fat mass, total body water, and fat-free mass) were monitored and reviewed regularly. Biochemical parameters—including lipid profile, markers of glucose metabolism, adiponectin, leptin, glucagon-like peptide-1 (GLP-1), and RMR—were assessed at baseline and after three months of dietary intervention. **Results**: Both diets had a beneficial effect on monitored parameters; however, the LGI diet was shown to be superior in reducing waist circumference, LDL, non-HDL cholesterol, leptin, and HbA1c, and in increasing GLP-1 levels without decline in RMR. **Conclusions**: A low-GI diet, under dietary supervision, can improve metabolic performance and promote weight reduction in people with obesity.

## 1. Introduction

Obesity is a complex chronic disease requiring a multifaceted therapeutic approach. The basic methods of obesity management include lifestyle modifications, pharmacotherapy, and bariatric surgery, which are combined to achieve optimal results [1]. Among lifestyle modifications, several nutrition interventions are recommended for treating obesity and improving metabolic health, including energy-restricted, Mediterranean, DASH, vegetarian, and low-glycemic index dietary patterns. Combining these diets with behavioral therapy and physical activity enhances their effectiveness and supports long-term weight management [1].

The glycemic index (GI) categorizes carbohydrate-containing foods based on their postprandial blood glucose response, with low-GI foods inducing slower and lower rises in blood glucose and insulin levels compared to high-GI diets [2]. This concept has gained attention as a dietary strategy for individuals with overweight and obesity [3,4,5]. The key mechanism by which low-GI diets may support weight management is through their influence on postprandial glucose concentration and insulin secretion, which may potentially reduce fat deposition and improve insulin sensitivity [6].

Randomized controlled trials (RCTs) and meta-analyses provide mixed but insightful results on the effects of low-GI diets on obesity management outcomes. In the GLYNDIET RCT study involving overweight and obese adults, the low-GI energy-restricted diet was more effective in BMI reduction. It also triggered more favorable changes in fasting insulin and insulin resistance markers than isocaloric high-GI and low-fat diets [7]. A systematic review involving 101 studies and more than 8500 participants concluded that a low-GI diet caused small but significant improvements in body weight and lipid profile (total cholesterol level), especially when the diet’s GI varied by at least 20 points [3]. A meta-analysis of 10 randomized clinical trials evaluating the effects of low-GI/glycemic load diets vs. high-GI/GL diets on body weight, fasting glucose, lipid profile, and insulin concentration revealed that individuals with obesity on low-GI/GL diets achieved greater body weight reductions, fasting glucose, and insulin levels than those on high-GI/GL diets. However, there were no intergroup differences in fat mass, waist circumference, and lipid profile [8]. A systematic review and meta-analysis of 24 randomized trials (2002 participants) showed that low-GI diets significantly reduced body weight, BMI, fasting blood glucose, and HbA1c concentration in patients with one of four common metabolic diseases (obesity, metabolic syndrome, diabetes, and cardiovascular disease) compared with control diets. Sustained adherence to low-GI can clinically improve weight management and glycemic control in metabolic diseases [9]. A recently published meta-analysis of six randomized controlled trials (192 adults, mean age 52.5 years) indicated that low-GI diets significantly reduced HOMA-IR scores compared with high-GI diets, highlighting their potential in improving insulin sensitivity in non-diabetic adults [10]. Additionally, according to Becker et al., the hypocaloric low-GI diet group showed greater reductions in body mass, BMI, percentage of body fat, waist-to-hip ratio, and leptin concentration compared to a control group of infertile women with excessive body weight. Moreover, a 12-week low-GI intervention has been shown to potentially improve oocyte development and pregnancy rates in women undergoing in vitro fertilization cycles [11].

On the other hand, observational data from 43 cohort studies (~1.94 million adults) and 30 meta-analyses of RCTs revealed no consistent associations between dietary GI and BMI or body fat, except when the GI difference exceeded 20 units in metabolically healthy adults. At the same time, the authors noted that nutrient density, dietary fiber intake, the proportion of whole grains, and the percentage of added sugar are more significant dietary characteristics in obesity therapy [5]. Evidence from a 2023 Cochrane review suggests that low-GI or low-GL diets are not significantly more effective at weight reduction for overweight or obese individuals than higher-GI/GL diets or other dietary approaches. Across 10 RCTs including over 1200 participants, these diets produced only small and clinically insignificant differences in body weight and body mass index. Similarly, effects on fat mass, fasting glucose, and other metabolic outcomes were minimal or uncertain [12].

Thus, the study aims to evaluate and compare the effects of a free-choice low-GI diet and a traditional low-energy diet in women with excessive body weight on the value of anthropometric parameters, selected indices of lipid, carbohydrate, and fat tissue metabolism, and alterations in resting energy expenditure.

## 2. Materials and Methods

### 2.1. Participants

Forty-seven women (age, 45.4 ± 9.4 years; range, 19–55 years) with overweight/obesity participated in the study. Participants were eligible for inclusion in the study if they met the following criteria: (1) BMI ≥ 25 and ≤40 kg/m^2^; (2) age between 18 and 55 years; (3) ability to provide informed consent; (3) regular menstrual cycles (no reported significant changes in cycle regularity), with no reported symptoms of menopause, no current use of hormone replacement therapy (HRT), lipid-lowering medications, or hypoglycemic agents; (4) no attempts to reduce body weight during the six months preceding the study; (5) no diagnosed chronic diseases, food allergies, or food intolerances; and (6) non-smokers. As part of the screening eligibility procedure, all participants underwent measurements of basic vital signs, including blood pressure, heart rate, body weight, and height. The screening was conducted by a physician. Participants had full access to all measurement results, and reporting and/or detecting any abnormalities at this stage resulted in exclusion from the study and a recommendation for medical consultation.

### 2.2. Study Design

The weight reduction program involved 46 overweight/obese adult women randomly assigned to either a low-GI diet (LGI) or a low-energy diet (LED). Participants were randomly assigned to the LED or LGI group using block randomization stratified by baseline BMI. Due to the nature of the dietary intervention (regular monitoring and consulting), blinding of participants and study staff was not possible.

Those assigned to the LGI group followed a diet based on a free choice of low-GI foods for 12 weeks: foods with a GI ≤ 55 could be consumed in quantities sufficient to ensure satiety, foods with a GI of 55–70 in quantities not exceeding two standard portions per day, and products with a GI > 70 were excluded from the diet. To make it easier to follow the diet, each participant received sample menus and materials with a list of foods categorized by their glycemic index. The protein and fat content remained unchanged, and the recommendations were similar to those for the second group. Those qualified for the LED group followed a traditional low-energy diet for 12 weeks with an energy deficit of 800 kcal/day, providing 25–30% of energy from fat, 20% from protein, and 50–55% from carbohydrates. Each participant received a sample menu to help them adhere to the diet. Participants remained under the care of a nutritionist during the entire study period.

The weight loss program included group and individual meetings conducted every two weeks. At the first meeting, information on dietary recommendations was provided, and during subsequent meetings, compliance with these recommendations was systematically monitored based on the patients’ daily food intake records. Nutritional education to improve healthy eating habits was also provided during the meetings. Patients were under the care of dietitians of the dietetic center at the Faculty of Human Nutrition of the Warsaw University of Life Sciences. Body weight, body composition (BIA), and waist circumference were measured during the biweekly meetings. At the beginning of the program and after 12 weeks, the following overnight fasting parameters were assessed: total cholesterol, LDL cholesterol, HDL, triglycerides, glucose, glycated hemoglobin (HbA1c), insulin, leptin, adiponectin, tumor necrosis factor alpha (TNF-α), and glucagon-like peptide 1 (GLP-1), concentrations in blood. Furthermore, at the beginning and end of the intervention (after 3 months), resting metabolic rate (RMR) was measured using indirect calorimetry. The study protocol complied with the Declaration of Helsinki, and the Bioethics Committee of the Institute of Food and Nutrition in Warsaw approved the study (KE/IŻŻ/29102009). All subjects signed a consent form to participate in the study.

### 2.3. Anthropometric Measurements

Weight and height measurements were taken without shoes, wearing light clothing, in a standing position, and with an upright posture, using a calibrated digital medical scale (Radwag, Radom, Poland), accurate to 0.1 kg, and a stadiometer (Radwag) with accuracy to 0.1 cm. Waist circumference was measured at the midpoint between the lowest rib and the iliac crest using a flexible, non-stretchable measuring tape, and recorded to the nearest 0.1 cm.

### 2.4. Body Composition Analysis

Body composition was evaluated using the AKERN RJ BIA 101/S bioelectrical impedance analyzer (AKERN Srl, Pontassieve, Italy) and Bodygram 3.1 software, in standard conditions. Participants were instructed to arrive fasted for measurements or at least 4 h after a meal, avoid strenuous exercise for 24 h, and empty their bladder before the measurement. The procedure was conducted according to the manufacturer’s guidelines. Parameters assessed included fat mass (FM), fat-free mass (FFM), and total body water (TBW).

### 2.5. Respirometry Measurements

Resting energy expenditure (RMR) was measured using indirect respirometry in all female subjects before the start of the study and after three months on the diet. To determine RMR under conditions of psychological and thermal comfort (constant room temperature of 22 ± 1 °C) for the participants in a semi-recumbent position, oxygen and exhaled carbon dioxide consumption were measured using a Fitmate Plus (Cosmed, Rome, Italy) apparatus and single-use respirometric masks. This measurement was performed on an empty stomach, after prior (no less than 5 min) adaptation to the conditions of the study, in order to achieve a coefficient of variance of measurements ≤ 10%. In addition, participants were asked to refrain from intense physical activity the day before the study and not smoke or take caffeinated beverages/products 12 h before the measurement.

### 2.6. Nutrition Assessment

The three-day food record method (dietary diaries) was used to assess the dietary intake of the study participants quantitatively. After appropriate training, the participants were asked to thoroughly record on a questionnaire the current consumption (real time) of the amount of products, beverages, and foods consumed for three non-consecutive days, each week of the study. To ensure the accuracy and completeness of the data collected, respondents were trained to properly record their food intake, including food specifications, serving sizes, preparation techniques, brand names of commercially available products, and detailed recipes for complex dishes. The Diet 5 program (Food and Nutrition Institute, Poland) was used to assess the energy and nutritional value of the menus. Glycemic index and glycemic load values were calculated using data from available tables [13]. The values of the index and glycemic load of foods that were not included in the tables were estimated based on the index data of the occurring products/foods most similar in composition and method of preparation.

### 2.7. Biochemical Analysis

The lipid profile was determined using standard enzymatic colorimetric assays with spectrophotometric detection. LDL cholesterol was calculated using the Friedewald formula (the formula was applied for TG values < 400 mg/dL). Non-HDL cholesterol concentration was calculated as the difference between total cholesterol and HDL cholesterol. Fasting plasma glucose concentration was measured by an enzymatic colorimetric method using glucose oxidase and spectrophotometric detection. Glycated hemoglobin (HbA1c) was defined using high-performance liquid chromatography (HPLC). Serum insulin was determined by radioimmunoassay. Insulin resistance was estimated using the HOMA-IR index, calculated as fasting insulin (µIU/mL) × fasting glucose (mg/dL)/405. Serum concentrations of leptin, adiponectin, and GLP-1 were measured using commercially available enzyme-linked immunosorbent assays (ELISA) according to the manufacturers’ instructions.

### 2.8. Statistical Analysis

Data were analyzed using Statistica software (StatSoft, Tulsa, OK, USA, version 13.3). Normality of distribution was assessed using the Shapiro–Wilk test. When assumptions of normality were met, group comparisons were made using the *t*-test for independent samples. Within-group changes over time were evaluated using the paired samples *t*-test. For non-normally distributed dependent variables, we used the Wilcoxon signed-rank test, and for the independent variables, the Mann–Whitney U test was used if this assumption was not fulfilled. As several variables did not meet the normality assumption, associations (strength and direction) between variables were evaluated using Spearman’s rank correlation coefficient (ρ). A significance level of *p* < 0.05 was assumed for all analyses.

## 3. Results

There were 65 women who volunteered for the study; 17 of them did not meet the inclusion criteria, and two dropped out before the study started. Therefore, the final number of participants was 46. Baseline characteristics of the study participants (in total and by group) are displayed in Table 1 and Table 2. There were no significant differences in the age and anthropometric indicators of the participants (Table 1).

No significant differences in the values of selected biochemical indicators and resting metabolic rate values were observed between the LED and LGI groups at baseline, indicating that the groups were homogeneous and comparable in terms of the presented parameters. The exception is the concentration of adiponectin, which was significantly higher in the LGI group than in the LED group (*p* < 0.001, *t*-test for independent variables) at the beginning of the study (Table 2).

As this difference in adiponectin concentration was present before the intervention, it likely reflects natural inter-individual variation rather than an effect of the dietary protocol. Several factors—such as differences in habitual dietary patterns or unmeasured lifestyle variables—may contribute to baseline adiponectin variability.

Additionally, none of the participants reported irregular menstrual cycles, a change in cycle length, or no menstruation in the past year during qualification, suggesting that none of the participants experienced menopause.

### 3.1. Dietary Intake

Data analysis showed statistically significant intergroup differences between both glycemic index and glycemic load values, dietary fiber intake, and percentage of polyunsaturated fatty acids (PUFA as % of total energy). The LGI group’s diet was characterized by a significantly lower glycemic index (Z = 3.95, *p* <0.001, Mann–Whitney U test) and glycemic load (*p* < 0.001, *t*-test for independent variables) and a higher intake of dietary fiber (Z = −2.08, *p* = 0.037, Mann–Whitney U test), PUFAs (Z = −2.56, *p* = 0.010, Mann–Whitney U test), and a higher polyunsaturated to saturated fatty acid (P:S) ratio (Z = −3.20, *p* = 0.0014, Mann–Whitney U test) (Table 3).

### 3.2. Anthropometric Indices

In both the LED and LGI groups, a considerable decline in body weight (*p* > 0.001, *t*-test for dependent variables), BMI (*p* > 0.001, *t*-test for dependent variables), fat mass (Z = 4.01, *p* > 0.001 and Z = 3.63, *p* = 0.0003, respectively, paired Wilcoxon signed-rank test), and waist circumference (Z = 4.19, *p* > 0.001 and Z = 4.10, *p* > 0.001, respectively, paired Wilcoxon signed-rank test) was recorded with a statistically significant increase in body water content (Z = 3.71, *p* > 0.001 and Z = 3.21, *p* = 0.0013, respectively, paired Wilcoxon signed-rank test). None of the groups showed significant differences in fat-free body mass after 12 weeks of diet compared to baseline values. Hence, to compare the size effect of the two diets on anthropometric parameters, the changes in magnitude (Δ) were analyzed. The boxplot presents the distribution of changes (Δ) in anthropometric parameters measured before and after the dietary intervention (end-baseline) (Table 4).

Following 12 weeks of dietary intervention, the LGI observed a significantly greater reduction (Δ) in waist circumference than the LED group (Z = 2.73, *p* < 0.006, Mann–Whitney U test). However, no significant between-group differences were detected for the other anthropometric parameters.

### 3.3. Lipid Profile

After 12 weeks, significant changes in blood lipid parameters were observed in both groups. In the LED group, total cholesterol (*p* = 0.04, *t*-test for dependent variables) and HDL cholesterol levels were significantly reduced (Z = 3.06, *p* = 0.0022, paired Wilcoxon signed-rank test). While in the LGI group, a significant decrease in total cholesterol (*p* = 0.0073, *t*-test for dependent variables), LDL (*p* = 0.0005, *t*-test for dependent variables), non-HDL cholesterol (*p* = 0.004, *t*-test for dependent variables), and triglyceride concentrations was observed (Z = 2.62, *p* = 0.009, paired Wilcoxon signed-rank test), with no substantial change in blood HDL levels compared to baseline values. This represents favorable changes in atherogenic lipid parameters. Table 5 shows a comparison of the magnitude of change in the given parameters expressed as the delta of change (baseline and end value) between the study groups (Table 5).

According to the Mann–Whitney U test, no significant difference was found between the LED and LGI groups regarding the change (Δ) in total cholesterol concentration. The reduction in the level of non-HDL cholesterol in blood was significantly greater in the LGI group compared with the LED group (Z = 2.25, *p* = 0.024). Moreover, *t*-tests (for independent variables) revealed a significantly greater reduction in LDL cholesterol in the LGI group than the LED group (*p* = 0.024). No significant between-group differences were observed in HDL cholesterol or triglyceride concentration changes.

### 3.4. Glucose Metabolism Parameters

After 12 weeks, significant changes in glucose metabolism parameters were observed in both groups. In the LED group, insulin concentrations and HOMA-IR values decreased significantly compared to baseline values (*t*-test for dependent variables, *p* < 0.05 for all). At the same time, in the LGI group, there was a significant decrease in insulin concentration. HbA1c, and HOMA-IR value (*t*-test for dependent variables, *p* < 0.05 for all). Table 6 shows a comparison of the magnitude of change in the given parameters expressed as the delta of change (baseline and end value) between the study groups.

Comparing the effect of the type of diet on the parameters of glucose metabolism, it was found that a significantly higher reduction in HbA1c concentration (Δ, end-baseline) was achieved on the LGI diet (*p* = 0.023, *t*-tests for independent variables). There were no significant differences between alterations in other parameters.

### 3.5. Fat Tissue Hormone Profile and GLP-1 Concentration

After 12 weeks, noteworthy changes in fat tissue hormones and GLP-1 concentration were recorded in both groups. A significant decrease in leptin concentrations was observed in both the LED and LGI groups (Z = 3.78, *p* < 0.001, and Z = 4.10, *p* < 0.001, respectively, paired Wilcoxon signed-rank test). In contrast, adiponectin (*t*-test for dependent variables, *p* < 0.001) and GLP-1 concentrations increased compared to values at the start of the study (Z = 4.26, *p* < 0.001 and Z = 4.01, *p* < 0.001, respectively, paired Wilcoxon signed-rank test). Table 7 shows a comparison of the magnitude of change in the given parameters expressed as the delta of change (baseline and end value) between the study groups.

A significant difference in GLP-1 change was observed between the LED and LGI groups (Mann–Whitney U = 141.5, Z = −2.68, *p* = 0.007). Participants in the LGI group demonstrated a greater increase in GLP-1 levels compared to the LED group. After 12 weeks of dietary intervention, the LGI group showed a greater decrease in leptin levels (*p* = 0.042, *t*-test for independent variables) than the LED group, while no significant differences were observed in adiponectin levels.

### 3.6. Resting Metabolic Rate

**Figure 1:**
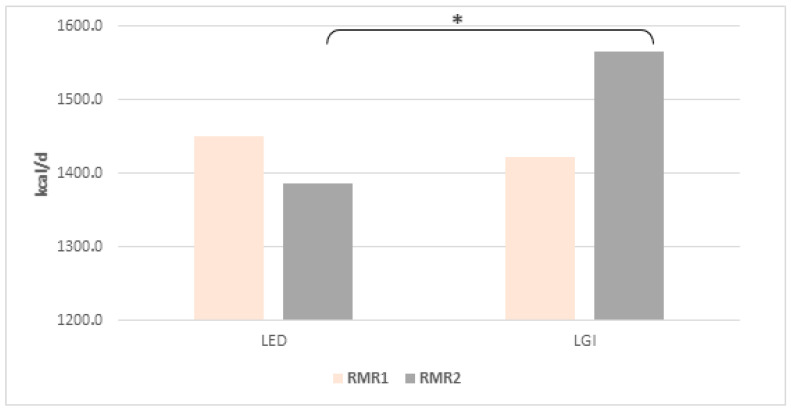
Mean (±SD) RMR value at the beginning (RMR1) and after 12 weeks (RMR2) of the diets; * indicates significant intergroup differences at *p* < 0.05.

**Figure 2:**
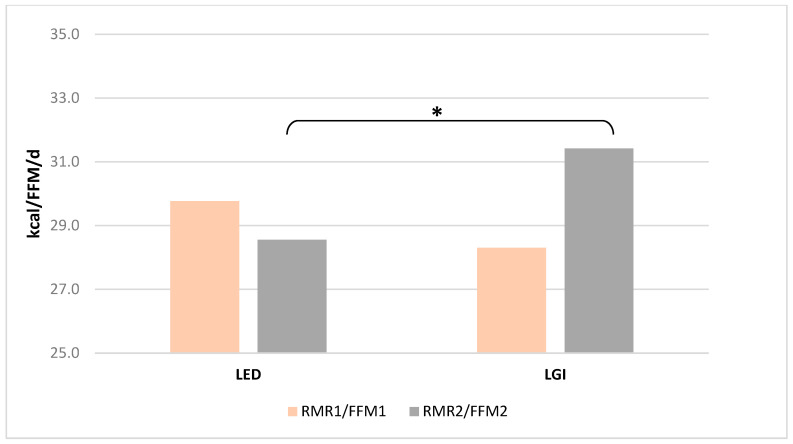
Mean (±SD) RMR/FFM value at the beginning (RMR1/FFM1) and after 12 weeks (RMR2/FFM2) of the diets; * indicates significant intergroup differences at *p* < 0.05.

Determined by indirect respirometry, the resting metabolism rate (RMR), both in its absolute value (Figure 1) and in relation to fat-free mass (FFM, kg) (Figure 2), did not differ significantly in women from both groups before the start of the study. However, after 12 weeks of LED adherence, RMR (Figure 1) and RMR/FFM (Figure 2) values were significantly lower than in women of the LGI group (*p* = 0.006 and *p* = 0.45, *t*-test for independent values, respectively). No statistically significant alterations in fat-free mass were detected in either group when comparing baseline measurements with those obtained after 12 weeks.

### 3.7. Selected Correlation

According to Spearman’s rank analysis of all results, a positive relationship has been demonstrated between changes in waist circumference and changes in body weight (Spearman’s ρ = 0.58, *p* < 0.05), BMI value (Spearman’s ρ = 0.57, *p* < 0.05), and fat body percentage (Spearman’s ρ = 0.50, *p* < 0.05).

Spearman’s rank correlation analysis of all data revealed a significant negative association between IG and Δ GLP-1 concentration (Spearman’s ρ = −0.39, *p* < 0.05) and adiponectin concentration after 12 weeks of dietary intervention (Spearman’s ρ = −0.49, *p* < 0.05). A positive relationship between fiber intake and adiponectin concentration at the end of the study (Spearman’s ρ = 0.49, *p* < 0.05) and a negative relationship between %PUFA and LDL-C concentration after the 12-week intervention (Spearman’s ρ = −0.52, *p* < 0.05).

In the LED group, a positive relationship was also recorded between Δ waist circumference, Δ triglyceride concentration (Spearman’s ρ = 0.55, *p* < 0.05), and Δ HOMA-IR value (Spearman’s ρ = 0.52, *p* < 0.05).

In turn, in the LGI group, data analysis also revealed a negative association between IG and adiponectin concentration at the end of the study (Spearman’s ρ = −0.48, *p* < 0.05) and Δ glucose concentration (Spearman’s ρ = 0.57, *p* < 0.05). Fiber intake in the LGI group was positively correlated with the Δ total cholesterol concentration (Spearman’s ρ = 0.46, *p* < 0.05) and GLP-1 concentration at the end of the study (Spearman’s ρ = 0.58, *p* < 0.05).

## 4. Discussion

The present study compared the effectiveness of two dietary models (low-glycemic index and low-energy diet) in reducing weight and their effects on cardiometabolic parameters and resting metabolic rate in women with excessive weight. Analysis of the results shows that the effectiveness of the diets differed in their impact on anthropometric parameters, metabolic parameters and energy expenditure. The observed variability suggests that not all diets exert an equivalent effect on metabolic health, and their effectiveness may depend on nutrient composition. Both diets followed by the participants appeared to have a similar energy value of 1100–1200 kcal/d, although in our study, the LGI diet was implemented without specific energy deficit guidelines or with caloric targets. The LGI diet was characterized by significantly lower GI and GL values, accompanied by a higher dietary fiber and PUFA intake.

The result of the 12-week LGI and LED diets was a significant comparable mean reduction in body weight (by 5.2 and 4.6 kg, respectively), BMI values (1.9 and 1.7 kg/m^2^, respectively), and body fat percentage (3.2 and 2.9% of body weight, respectively) in comparison to the initial values. However, a significantly greater reduction in waist circumference was noted in women following the LGI diet. This is beneficial from a health perspective, as waist circumference is a key indicator of abdominal obesity, and it is strongly associated with cardiometabolic disorders: hypertension, type 2 diabetes mellitus, dyslipidemia, and coronary heart disease [14].

The evidence from a systematic review and meta-analysis of randomized controlled trials comparing the effects of a low-glycemic index diet and other types of diets (high GI, low fat, a routine diet, etc.) on the body weight and BMI value in patients with four common metabolic diseases (obesity, metabolic syndrome, diabetes, CVD), shows that low-GI diets produced modest but more pronounced improvements in anthropometric parameters compared with control diets in individuals with metabolic disorders [9]. The meta-analysis demonstrated a mean reduction in body weight of approximately 2.6 kg (95% CI: −4.35 to −0.95) and a decrease in BMI of about 0.7 kg/m^2^ (95% CI: −1.18 to −0.27). When interventions lasted 24 weeks or longer, the reduction in BMI became more pronounced, and study heterogeneity markedly decreased [9]. In contrast, another summary of studies comparing the effect of low glycemic index or low glycemic load diets with high GI/LG diets in individuals with an excessive body mass showed that low GI/LG diets did not result in significantly greater weight reductions in the overweight population (BMI ≥ 25 kg/m^2^). However, in obese participants (BMI ≥ 30 kg/m^2^), low-GI/GL diets led to a small but significant additional weight loss of about 0.9 kg compared to high-GI/GL diets. No differences were observed in fat mass, fat-free mass, waist circumference, or lipid profile between low-GI/GL and high-GI/GL diets [8]. In turn, in a large prospective cohort study conducted in five European countries, the association between the diet glycemic index and glycemic load and subsequent changes in body weight and waist circumference was examined in almost 90,000 adults. During an over ten-year follow-up period, higher diet GI and GL were not significantly associated with overall weight change, while higher GI was marginally associated with an increase in waist circumference (0.19 cm per year for every 10-unit increase in GI). These results suggest that although overall body weight may remain unaffected, a diet with a lower GI may contribute to reducing the risk of abdominal obesity [15], potentially influencing the insulin sensitivity of visceral fat. A similar conclusion can be drawn based on the results obtained from this analysis. where women following the LGI diet showed a significantly greater reduction in waist circumference compared to the LED group. Waist circumference is a critical component in diagnosing metabolic syndrome; thus, its notable reduction can lead to significant improvements in metabolic parameters with special emphasis on enhanced insulin sensitivity [16].

This was also observed in our study: changes in anthropometric parameters triggered changes in metabolic parameters. The reduction in the LDL-C and non-HDL cholesterol concentration was significantly greater in the LGI group compared with the LED group. Similar results were observed across 28 randomized controlled trials (1.272 participants), showing that low glycemic index diets led to small but significant reductions in total cholesterol (−0.13 mmol/L) and LDL cholesterol (−0.16 mmol/L). The effects were stronger with greater reductions in dietary GI and higher fiber intake [17]. The LGI group in our study similarly showed a significant relationship between dietary fiber intake and changes in total cholesterol levels; moreover, a significantly higher proportion of dietary fiber in this group (than in the LED) may have contributed to changes in LDL-C and non-HDL-C levels. In addition, this effect may also have been attributed to the statistically higher PUFA content of the LGI and significantly higher P:S ratio than in the LED group (median values 0.7 vs. 0.5, respectively). Since a meta-analysis of 24 randomized trials (1011 participants) showed that a diet with a higher ratio of polyunsaturated to saturated fatty acids (median P:S ratio of 1.2) significantly lowered LDL cholesterol (−9.8 mg/dL) compared to a diet with a lower P:S ratio (median P:S ratio of 0.4) [18]. Thus, it can be assumed that an effective strategy for lowering LDL levels is the substitution of saturated fats for polyunsaturated fats to increase the P:S diet ratio.

Our observations show that both diets have a beneficial effect on glucose metabolism parameters, including a reduction in fasting insulin concentration and HOMA-IR, resulting from changes in anthropometric parameters, mainly a reduction in body fat and waist circumference. However, after 12 weeks, only the LGI diet led to a significant reduction in HbA1c values compared to the LED diet. Multiple studies indicate that low-GI diets improve glycemic control as evidenced by significant reductions in fasting blood glucose, insulin, and HbA1c levels [9,19]. A meta-analysis of the data showed that low-GI diets were generally more effective than other diets in reducing HbA1c when the intervention lasted 24 weeks or longer, although the difference was not statistically significant [9].

After a 12-week dietary intervention program, significant changes in adipokines were also observed, with a significant reduction in leptin concentration (more pronounced in the LGI group) and a simultaneous increase in blood adiponectin and GLP-1 levels (more pronounced in the LGI group). As the glycemic load of both diets does not exceed 100, they can be considered low-glycemic-load diets. Thus, the results obtained in our study regarding adiponectin are similar to the results obtained by Neuhouser et al. [20]. In a study (RCT), authors evaluated the effect of a low glycemic load diet on inflammatory biomarkers and adiposity in normal-weight and overweight/obese adults. After the controlled intervention period, in participants with high body fat, the low-GL diet reduced C-reactive protein and increased adiponectin compared to the high-GL diet. These results suggest that a low glycemic load diet may beneficially modulate inflammation and adipokine profile, potentially reducing the risk of obesity-related complications [20]. The increase in adiponectin (both groups) and decrease in leptin levels can be partially explained in the LGI group by significantly higher PUFA and fiber content. Studies have shown that omega-3 fatty acids can activate peroxisome proliferator-activated receptor gamma (PPARγ), thereby promoting adiponectin synthesis in adipose tissue [21]. In turn, according to Qi et al. (2006) [22], higher dietary fiber intake was positively associated with plasma adiponectin concentrations, and a higher dietary glycemic load was linked to lower adiponectin levels. These relationships remained prominent even after adjustment for obesity and other confounders, suggesting that both greater fiber intake and lower glycemic load may beneficially influence adiponectin levels in women with type 2 diabetes [22].

In addition, the increase in adiponectin concentrations (concerning the baseline values and comparable in both groups) can be explained (partially) by the reduction in body weight, mainly body fat content, in both groups. Previous studies have demonstrated that individuals with obesity exhibit reduced circulating adiponectin levels, while weight reduction results in an increase in adiponectin plasma concentration (Yang et al., 2001; Ma et al., 2016 [23,24]). This increase is considered one of the mechanisms linking weight reduction to improved metabolic health (Kishida et al., 2012 [25]). Therefore, assessing adiponectin dynamics before and after an intervention provides valuable insight into the cardiometabolic benefits associated with weight reduction. Moreover, the results of our study are consistent with data showing that modifying the glycemic response through diet can significantly affect metabolic and hormonal regulation. In particular, a randomized controlled trial conducted by Becker et al. [11] showed that short-term (also 12-week) adherence to a low-calorie, low glycemic index and load diet resulted in a significant reduction in BMI and body fat in overweight and obese women with infertility compared to a control diet. Interestingly, leptin concentrations decreased significantly despite only moderate weight loss. These results support our observations that a hypocaloric diet with an adequate quantity and quality of carbohydrates can act as a modulator of metabolic parameters.

Although both diets positively affected GLP-1 concentration, which is involved in improved glucose regulation and appetite control, participants in the LGI group demonstrated a greater increase in GLP-1 levels compared to the LED group. Data indicate that short-chain fatty acids (SFAs), produced during fiber fermentation by the gut microbiota, can stimulate GLP-1 secretion [26]. Only in the LGI group, fiber intake positively correlates with GLP-1 concentration at the end of the study, which might partially explain the observed effect.

In the LGI group, no decrease in RMR accompanied by weight reduction was observed. These results contrast with findings reported by Karl et al. [27], which evaluated the effects of diets with different carbohydrate content (moderate 55% or high 70%) and different glycemic indexes (low or high) on body composition and resting metabolic rate during weight loss in obese adults. The study involving 91 subjects showed that neither the carbohydrate content nor the GI value of the diet affected total weight loss, the amount of fat mass decreased, or the maintenance of lean body mass. In addition, the metabolic adaptation observed (a decrease in RMR greater than predicted) was not differentiated by diet composition and apparently disappeared after a period of weight stabilization. The researchers concluded that moderate-carbohydrate and low-GI diets do not confer preferential metabolic benefits over high-carbohydrate and high-GI diets when other dietary factors, such as protein, are tightly controlled [27]. Another study has demonstrated that the decrease in resting and total energy expenditure, compared to baseline values before weight loss, was greatest on the low-fat diet, medium on the low-glycemic-index diet, and least on the very-low-carbohydrate diet, suggesting that carbohydrate quantity, rather than glycemic index, plays a more significant role in sustaining energy expenditure following weight loss [28]. A low glycemic index diet can promote favorable fat mass reduction and maintenance of fat-free body mass during weight loss, as we did not observe variation in FFM compared to baseline values. Thus, preserving fat-free mass during weight loss is important for maintaining metabolic rate, muscle function, and long-term weight control [29]. Nevertheless, the LGI group showed a smaller reduction in resting metabolism; this observation may have been influenced by variability in baseline values and differences in energy intake. Since these factors were not adjusted, the results should be considered descriptive and not indicative of an actual protective effect on RMR. Further studies specifically designed and powered to assess changes in RMR are required to confirm this observation.

In our opinion, a strength of the study was a well-planned dietary intervention with professional support. Regular contact with a dietitian and consistent monitoring of dietary adherence contributed substantially to the program’s effectiveness in reducing body weight, decreasing waist circumference, and improving cardiometabolic parameters. These assumptions are in line with previous evidence showing that professional counseling and structured collaborative activities with the patient increase the effectiveness and sustainability of weight loss interventions [30]. A broad panel of analyses (anthropometric, biochemical, and RMR examinations) provides a comprehensive picture of the intervention’s effects and demonstrates its practical application. However, the relatively small sample size, follow-up time, and completion of a diet diary may limit the generalizability and long-term interpretation of the results. Potential confounders, including variations in fiber and PUFA intake as well as differences in diet adherence, may have also influenced the observed outcomes. Multiple unadjusted comparisons represent a limitation and call for cautious interpretation of the findings. Despite these limitations, the study’s findings provide valuable information on effective obesity treatment strategies and highlight the importance of ongoing professional dietary counseling in this area.

## 5. Conclusions

Overall, both energy-restricted diets have proved to be an effective tool in managing obesity and improving cardiometabolic health by enhancing insulin sensitivity, improving the lipid profile, and favorably modulating hormones like adiponectin, leptin, and GLP-1, which, in addition, was not accompanied by a decrease in fat-free mass. Nevertheless, the LGI diet was shown to be more beneficial in reducing waist circumference, LDL, non-HDL cholesterol, leptin, and HbA1c, and in increasing GLP-1 levels. Furthermore, in this group, no decline in resting metabolic rate was noted. Although those data suggest superior effects of the LGI diet, these findings are exploratory and should be interpreted with caution due to the limited sample size and lack of blinding.

## Figures and Tables

**Table 1:** Age and anthropometric data of study participants at baseline.

Parameter	Means ± SD			Median (Min–Max)	
	All (N = 46)	LED (n = 24)	LGI (n = 22)	LED (n = 24)	LGI (n = 22)
Age (y)	45.1 ± 9.5	44.2 ± 10.2	46.2 ± 8.9	48 (19.0–55.0) †	48.5 (22.0–55.0) †
Body mass (kg)	84.5 ± 11.1	85.0 ±11.0	83.8 ± 11.6	82.0 (68.0–103.0)	82.5 (66.2–104.3)
BMI (kg/m^2^)	31.7 ± 3.9	32.1 ± 4.0	31.3 ± 4.0	31.1 (26.3–41.5)	30.9 (25.3–40.7)
FM (%)	41.2 ± 4.6	42.1 ± 5.1	40.2 ± 4.0	42.7 (30.5–49.3)	41.6 (31.4–46.8)
FFM (kg)	49.5 ± 5.1	48.9 ± 5.2	50.1 ± 5.1	48.0 (37.5–60.1)	51.2 (40.4–57.2)
TBW (%)	42.7 ± 3.9	42.6 ± 3.7	42.8 ± 4.2	42.1 (37.1–50.8)	42.6 (31.2–50.6)
WC (cm)	99.3 ± 10.0	96.8 ± 10.0	102.0 ± 9.4	98.0 (78.0–118.0)	102.8 (87.5–125.0)

BMI—basal metabolic rate; FM—fat mass; FFM—fat-free mass; TBW—total body water; WC—waist circumference; †—variable with non-normal distribution (Shapiro–Wilk *p* ≤ 0.05); median and min-max range are shown; non-parametric test was used.

**Table 2:** Plasma parameters and RMR of study participants at baseline.

Parameters	Means ± SD			Median (Min–Max)	
	All (N = 46)	LED (n = 24)	LGI (n = 22)	LED (n = 24)	LGI (n = 22)
TC (mg/dL)	213.2 ± 43.8	211.9 ± 45.1	214.6 ± 43.4	211.1 (138.9–290.7)	211.7 (152.6–328.9)
LDL-C (mg/dL)	123.5 ±34.6	126.5 ± 37.1	120.2 ± 32.0	127.5 (69.0–191.0)	118.0 (74.4–189.0)
HDL-C (mg/dL)	64.9 ± 17.8	62.6 ± 16.6	67.4 ± 19.1	59.8 (41.1–123.7) †	69.3 (36.9–109.0)
Non-HDL-C (mg/dL)	148.3 ± 39.3	149.3 ± 38.0	147.2 ± 41.6	147.8 (94.6–219.9)	141.3 (86.4–261.0)
TG (mg/dL)	113.9 ± 44.8	113.5 ± 44.5	114.4 ± 46.2	114.8 (38.2–182.5)	110.3 (40.6–208.5)
Glucose (mg/dL)	85.0 ± 8.8	83.6 ± 8.3	86.5 ± 9.3	81.4 (71.2–100.5)	84.1 (71.0–101.8)
HbA1c (%)	5.7 ± 0.7	5.6 ± 0.7	5.9 ± 0.7	5.7 (4.4–6.6)	6.1 (4.5–6.8)
Insulin (µUI/mL)	10.0 ± 3.6	10.0 ± 3.8	10.0 ± 3.5	8.5 (4.8–19.1)	9.5 (3.4–16.4)
HOMA-IR	2.1 ±0.8	2.1 ± 0.8	2.1 ± 0.8	1.8 (1.0–3.7)	2.1 (0.6–4.0)
Adiponectin (µg/mL)	12.6 ± 3.6	10.7 ± 3.0 *	14.7 ± 2.9 *	10.3 (2.9–18.2)	15.3 (9.8–21.9)
Leptin (ng/mL)	28.4 ± 11.2	27.4 ± 10.3	29.5 ± 12.3	26.1 (7.9–50.0)	26.5 (8.3–50.0)
GLP-1 (ng/L)	0.7 ± 0.3	0.72 ± 0.3	0.7 ± 0.4	0.7 (0.3–1.5) †	0.6 (0.1–2.0) †
RMR (kcal/d)	1436.5 ± 291.1	1450.1 ± 235.4	1421.6 ± 347.1	1487.0 (944.0–1883.0)	1460.5 (849.0–1958.0)

TC—total cholesterol; LDL-C—low-density cholesterol; HDL-C—high-density cholesterol; TG—triglycerides; HbA1c—glycosylated hemoglobin; GLP-1—glucagon-like peptide 1; TNF-α—tumor necrosis factor α; RMR—resting metabolic rate; * indicates significant intergroup differences at *p* < 0.05; †—variable with non-normal distribution (Shapiro–Wilk *p* ≤ 0.05); median and min-max range are shown; non-parametric test was used.

**Table 3:** Nutritional value of the studied diets.

Indices	Means ± SD		Median (Min–Max)	
	LED (n = 24)	LGI (n = 22)	LED (n = 24)	LGI (n = 22)
GI	52.1 ± 5.1	44.1 ± 6.4	51.3 * (43.6–66.2)	43.0 * (36.4–58.3) †
GL	81.2 ± 17.8 *	56.0 ± 13.1 *	80.5 (46.6–119.3)	55.2 (32.1–85.1)
Energy (kcal/d)	1282.5 ± 180.9	1253.4 ± 133.7	1249.4 (1108.1–1577.0)	1237.2 (1112.1–1519.5)
Protein (% energy)	26.0 ± 6.4	25.9 ± 4.8	26.5 (13.1–41.6)	25.7 (18.5–34.9)
Fat (% energy)	24.0 ± 4.4	25.6 ± 7.4	22.9 (18.3–39.4) †	25.0 (15.6–47.4)
SFA (% energy)	9.9 ± 2.5	8.6 ± 2.4	8.8 (6.7–14.9) †	8.4 (5.7–15.4) †
PUFA (% energy)	4.6 ± 1.9	5.9 ± 2.3	4.2 * (2.3–11.8) †	5.7 * (2.7–12.3) †
P:S ratio	0.5 ± 0.2	0.7 ± 0.3	0.5 * (0.2–1.3) †	0.7 * (0.3–1.8) †
Carbohydrates (% energy)	50.4 ± 5.8	48.5 ± 6.3	50.6 (36.5–61.8)	48.8 (30.5–59.0)
Dietary fiber (g/d)	19.0 ± 6.9	21.8 ± 6.4	16.5 * (9.4–41.9) †	20.8 * (7.8–35.8)
Sodium (mg/d)	1658.4 ± 607.0	1298.2 ± 570.0	1564.8 (862.9–3595.0) †	1243.7 (379.4–2295.5)
Potassium (mg/d)	2789.4 ± 635.7	2860.8 ± 757.7	2610.0 (1843.3–4233.0)	2684.6 (1830.0–4512.8)
Calcium (mg/d)	575.6 ± 203.0	575.1 ± 222.3	528.1 (229.5–942.1)	523.3 (220.7–1210.7) †
Magnesium (mg/d)	259.9 ± 64.5	295.0 ± 91.9	250.1 (175.3–466.9) †	271.5 (179.8–508.4)
Iron (mg/d)	8.6 ± 1.5	9.3 ± 2.4	8.2 (6.8–12.2) †	8.5 (5.5–14.3) †
Zinc (mg/d)	8.2 ± 1.4	8.4 ± 2.1	8.2 (4.4–11.7)	7.4 (5.1–12.7) †
B1 vitamin (mg/d)	1.0 ± 0.3	1.1 ± 0.2	1.0 (0.5–1.5)	1.1 (0.7–1.6)
B2 (mg/d)	1.3 ± 0.3	1.5 ± 0.5	1.3 (0.8–2.0)	1.3 (0.8–2.9) †
B6 (mg/d)	1.5 ± 0.4	1.6 ± 0.5	1.5 (0.8–2.4)	1.5 (0.9–2.4) †
Folates (µg/d)	255.7 ± 64.5	281.9 ± 103.2	241.2 (157.8–438.1)	251.0 (161.4–541.3) †
B12 (µg/d)	3.0 ± 1.3	3.2 ± 2.3	3.0 (1.1–6.7)	2.3 (1.0–10.7) †
C vitamin (mg/d)	104.9 ± 80.6	105.1 ± 45.1	83.6 (21.7–302.9) †	111.6 (26.2–190.7)

GI—glycemic index; GL—glycemic load; SFA—saturated fatty acids; PUFA—polyunsaturated fatty acids; P:S ratio—polyunsaturated to saturated fatty acids ratio; * indicates significant intergroup differences at *p* < 0.05; †—variable with non-normal distribution (Shapiro–Wilk *p* ≤ 0.05); median and min-max range are shown; non-parametric test was used.

**Table 4:** Comparison of changes in values of anthropometric parameters at the beginning and end of the study (Δ, end-baseline) in each group.

Indices	Means ± SD		Median (Min–Max)	
	LED (n = 24)	LGI (n = 22)	LED (n = 24)	LGI (n = 22)
Δ Body weight (kg)	−4.6 ± 3.3	−5.2 ± 2.8	−4.3 (−10.6–0.2)	−5.2 (−12.1–−0.7)
Δ BMI (kg/m^2^)	−1.7 ± 1.2	−1.9 ± 1.1	−1.7 (−4.1–0.1)	−2.0 (−4.7–−0.3)
Δ FM (% body weight)	−2.9 ± 2.1	−3.2 ± 2.6	−2.9 (−6.0–1.5)	−3.6 (−6.8–4.5)
Δ TBW (% body weight)	1.9 ± 1.7	3.3 ± 4.9	2.2 (−1.4–4.5)	2.7 (−3.3–6.7) †
Δ FFM (kg)	−0.2 ± 1.6	0.4 ± 5.4	−0.3 (−4.6–3.0)	−0.5 (−5.5–2.0) †
Δ WC (cm)	−4.5 ± 3.3	−7.9 ± 5.5	−4.0 (−13.0–1.0)	−6.3 (−27.5–−1.0) *†

* indicates significant intergroup differences at *p* < 0.05; †—variable with non-normal distribution (Shapiro–Wilk *p* ≤ 0.05); median and min-max range are shown; non-parametric test was used.

**Table 5:** Changes in lipid profile parameters as Δ (end-baseline) in the LED (n = 24) and LGI (n = 22) groups after 12 weeks of dietary intervention.

Indices	Δ TC [mg/dL]		Δ LDL-C [mg/dL]		Δ HDL-C [mg/dL]		Δ Non-HDL-C [mg/dL]		Δ TG [mg/dL]	
Group	LED	LGI	LED	LGI	LED	LGI	LED	LGI	LED	LGI
Mean	−10.8	−21.6	−1.9 *	−13.9 *	−5.0	−1.0	−5.8	−20.6	−19.4	−12.2
Median	−3.8	−15.9 †	−0.5	−13.5	−5.9	−1.1	−3.2 *	−18.4 *†	−19.2	−10.0
Min	−77.1	−151.8	−59.0	−41.0	−19.5	−16.0	−67.5	−135.8	−96.1	−79.3
Max	25.6	16.7	34.0	17.0	14.3	12.8	24.6	15.1	63.3	64.2
SD	24.8	34.1	18.8	15.7	7.4	8.0	20.2	29.7	42.1	29.8

* indicates significant intergroup differences at *p* < 0.05; †—variable with non-normal distribution (Shapiro–Wilk *p* ≤ 0.05); median and min-max range are shown; non-parametric test was used.

**Table 6:** Changes in glucose metabolism parameters as Δ (end-baseline) in the LED (n = 24) and LGI (n = 22) groups after 12 weeks of dietary intervention.

Indices	Δ Gucose [mg/dL]		Δ HbA1c [%]		Δ Insulin [µUI/mL]		Δ HOMA-IR	
Group	LED	LGI	LED	LGI	LED	LGI	LED	LGI
Mean	1.84	−1.02	0.09	−0.28 *	−2.02	−1.68	−0.39	−0.36
Median	1.25	−1.20	0.05	−0.24	−2.65 †	−1.90	−0.46 †	−0.47
Min	−12.90	−15.40	−0.83	−1.42	−5.80	−7.50	−1.32	−1.81
Max	17.20	12.70	0.96	0.57	6.50	5.80	1.34	1.24
SD	7.00	8.87	0.56	0.47	2.79	3.70	0.62	0.82

* indicates significant intergroup differences at *p* < 0.05; †—variable with non-normal distribution (Shapiro–Wilk *p* ≤ 0.05); median and min-max range are shown; non-parametric test was used.

**Table 7:** Changes in leptin, adiponectin, and GLP-1 concentrations Δ (end-baseline) in the LED (n = 24) and LGI (n = 22) groups after 12 weeks of dietary intervention.

Indices	Δ Leptin [ng/mL]		Δ Adiponectin [µg/mL]		Δ GLP-1 [ng/L]	
Group	LED	LGI	LED	LGI	LED	LGI
Mean	−6.97 *	−11.05 *	1.76	1.86	0.74	1.38
Median	−8.15	−9.65	1.90	1.75	0.58	1.05 *†
Min	−17.45	−27.10	−1.60	−1.20	−0.10	0.14
Max	6.10	−0.80	6.80	5.79	1.78	4.49
SD	5.88	7.33	2.28	1.77	0.47	0.96

* indicates significant intergroup differences at *p* < 0.05; †—variable with non-normal distribution (Shapiro–Wilk *p* ≤ 0.05); median and min-max range are shown; non-parametric test was used.

## Data Availability

The original contributions presented in this study are included in the article. Further inquiries can be directed to the corresponding author.
